# Transcutaneous Spinal Neuromodulation Reorganizes Neural Networks in Patients with Cerebral Palsy

**DOI:** 10.1007/s13311-021-01087-6

**Published:** 2021-07-09

**Authors:** Parag Gad, Susan Hastings, Hui Zhong, Gaurav Seth, Sachin Kandhari, V. Reggie Edgerton

**Affiliations:** 1 Department of Neurobiology, University of California, Los Angeles, CA 90095 USA; 2 Rancho Research Institute, Downey, CA 90242 USA; 3 SpineX Inc, Los Angeles, CA 91324 USA; 4 Susan Hastings Pediatric Physical Therapy, San Jose, CA 95125 USA; 5 Indian Institute of Technology, Banaras Hindu University, Uttar Pradesh, Varanasi, 221005 India; 6 Institute of Brain and Spine, New Delhi, 110024 India; 7 Department of Neurosurgery, University of California, Los Angeles, CA 90095 USA; 8 Brain Research Institute, University of California, Los Angeles, CA 90095 USA; 9 Institut Guttmann, Hospital de Neurorehabilitació, Institut Universitari Adscrit a La Universitat Autònoma de Barcelona, 08916 Badalona Barcelona, Spain

## Abstract

Spinal neuromodulation and activity-based rehabilitation triggers neural network reorganization and enhances sensory-motor performances involving the lower limbs, the trunk, and the upper limbs. This study reports the acute effects of Transcutaneous Electrical Spinal Cord Neuromodulation (SCONE™, SpineX Inc.) on 12 individuals (ages 2 to 50) diagnosed with cerebral palsy (CP) with Gross Motor Function Classification Scale (GMFCS) levels ranging from I to V. Acute spinal neuromodulation improved the postural and locomotor abilities in 11 out of the 12 patients including the ability to generate bilateral weight bearing stepping in a 2-year-old (GMFCS level IV) who was unable to step. In addition, we observed independent head-control and weight bearing standing with stimulation in a 10-year-old and a 4-year old (GMFCS level V) who were unable to hold their head up or stand without support in the absence of stimulation. All patients significantly improved in coordination of flexor and extensor motor pools and inter and intralimb joint angles while stepping on a treadmill. While it is assumed that the etiologies of the disruptive functions of CP are associated with an injury to the supraspinal networks, these data are consistent with the hypothesis that spinal neuromodulation and functionally focused activity-based therapies can form a functionally improved chronic state of reorganization of the spinal-supraspinal connectivity. We further suggest that the level of reorganization of spinal-supraspinal connectivity with neuromodulation contributed to improved locomotion by improving the coordination patterns of flexor and extensor muscles by modulating the amplitude and firing patterns of EMG burst during stepping.

**Supplementary Information:**

The online version contains supplementary material available at 10.1007/s13311-021-01087-6.

## Introduction

Cerebral palsy (CP) is one of the most common childhood motor disorders in the USA with an estimated 1.5 to 4 out of 1000 individuals born with CP [1–3] and over 10,000 new cases diagnosed each year. There are approximately 500,000 children under the age of 18 in USA and approximately 17 million globally currently living with CP. Medical costs are around 10 times higher for children with CP and 26 times higher for children with CP with an intellectual disability. The total lifetime care costs currently exceed $1 million [4]. CP is generally considered to be an umbrella diagnosis that includes a wide range of symptoms with heterogeneous etiologies of neural and cardiovascular origins [5]. It is generally assumed that the primary pathology of the nervous system that leads to CP is located within and among different combinations of supraspinal networks, and these pathologies can be due to multiple etiologies. In most cases, however, it appears that these supraspinal pathologies also will be necessarily manifested as spinally mediated dysfunctions, affecting multiple peripheral sensory-motor systems including midline orientation, equilibrium, posture (including trunk and head control), locomotion, and trunk and head control [6]. Also, the visual system often is impacted in children with CP, because normally, the brain and spinal networks provide a key, coherent driving factor to accommodate a 1G environment in controlling posture. But children with CP often learn faulty trunk and head alignment as a result of the dysfunctional input from the supraspinal networks to the largely normal spinal networks that can be driven by the proprioceptive system [7].

The majority of children classified as Gross Motor Function Classification Scale (GMFCS) Level I are expected to reach their motor potential between 7 and 9 years and thereafter remain stable until age 21 when they may experience functional decline due to pain, weakness, and stiffness [8, 9]. Presently, all available interventions are designed to manage and minimize symptoms rather than correcting the underlying dysfunctions [10, 11]. While some commonly preferred treatments such as intramuscular injection of onabotulinumtoxinA (BotoxA) reduce symptoms of spasticity, which initially may improve function, but rarely lead to significant long-term functional changes [12] and may severely limit the eventual level of recovery that theoretically is possible [13].

We have demonstrated that noninvasive stimulation of the spinal cord can lead to recovery of lower extremity [14, 15], upper extremity [16], trunk [17], breathing and coughing [18], and bladder function [19, 20] after spinal cord injury (SCI). Some features of the physiological dysfunctions seen in individuals with SCI are similar to those commonly observed in individuals with CP, i.e., poor coordination of motor pools during stepping and spasticity. Multiple neuromodulation modalities have been attempted in children with CP for recovery of sensorimotor function including combined locomotor therapy and functional electrical stimulation [21, 22] and epidural [23, 24], but transcutaneous spinal stimulation [25], similar to that used in the present study, has been shown to have a greater effect. In the present study, we have merged ideas and concepts derived from both recent clinical observations of CP and decades of studies in SCI patients [16, 26–28] including activity-based mechanisms during locomotor tasks and transcutaneous spinal neuromodulation [15, 27, 29]. We *hypothesized* that a single (acute) transcutaneous spinal neuromodulation session could facilitate locomotor function of individuals with CP by reorganizing spinal neural networks to functional states which enable spinal interneurons to translate ensembles of proprioception to temporal patterns of activity that generates highly coordinated movements. How electrical neuromodulation transforms the networks to functional states which enable them to generate these improved coordinated patterns remains unknown, but the present observations provide a very important target given its significance to the level of plasticity of spinal and supraspinal networks of individuals with CP. Thus, the objective of this study is to determine whether the acute, system-level physiological effects of transcutaneous spinal cord neuromodulation has the potential to improve locomotor function in individuals with CP as has been shown to be effective in individuals with spinal cord injuries.

## Methods

The study was approved by an external Investigational Review Board (Integreview IRB). All study participants (or their parents in case of a minor) signed the informed consent form and consented to their data to be used for future publications and presentations. The patient demographic and pathologies are summarized in Table 1. The inclusion criteria included (1) individuals above the age of 2 years of age and (2) diagnosed with cerebral palsy (CP). The exclusion criteria included (1) selective dorsal root rhizotomy, (2) intramuscular Botox injection in the preceding 12 months, (3) current antispastic medications, (4) unhealed fractures or contractures that would prevent them from performing functional tasks, and (5) tendon-lengthening surgeries.

**Table 1:** Summary of the patient demographic and severity of the pathology

Pt. ID	Age (years)	Gender	Pathology	GMFCS level	Parameters T11/L1
P1	2.66	M	CP spastic quadriplegia	IV	15 mA/20 mA
P2	6.25	M	CP L spastic hemiplegia	II	30 mA/40 mA
P3	14	M	CP spastic diplegia	II	40 mA/50 mA
P4	50	F	CP spastic diplegia	I	40 mA/50 mA
P5	2.25	M	CP spastic quadriplegia	IV	20 mA/30 mA
P6	16	F	CP spastic quadriplegia—post hemispherectomy	I	40 mA/50 mA
P7	10	F	CP-dyskinetic quadriplegia	V	40 mA/50 mA
P8	4	M	CP spastic diplegia	II	25 mA/30 mA
P9	3	M	CP L spastic quadriplegia—post hemispherectomy	I	30 mA/30 mA
P10	3	M	CP spastic quadriplegia	V	30 mA/40 mA
P11	3	M	CP R spastic quadriplegia—post hemispherectomy	I	30 mA/45 mA
P12	4	M	CP-spastic triplegia	II	30 mA/40 mA

### SCONE™ Therapy

Spinal stimulation was delivered over the course of a single 30-min session using a proprietary device *SCONE™* (SpineX Inc, Los Angeles, CA) (Parag Gad 2019). The stimulation waveform consisted of two alternating pulses of opposite polarities separated by a 1-µS delay to form a delayed biphasic waveform. The pulses consisted of a high-frequency biphasic carrier pulse (10 kHz) combined with a low-frequency (30 Hz) burst pulse each with a pulse width of 1 ms. Stimulation was applied using an adhesive hydrogel electrode between T11–12 and L1–2 serving as the cathode and two adhesive hydrogel electrodes over bilateral iliac crests as the anodes (Supplemental Fig. 1). Patients were assessed both without and with neuromodulation at different parameters to test the changes in stepping patterns. However, at all times, the stimulation intensity was maintained at ~ 20–25% below the threshold that induced a motor response [15, 17, 29–31]. All patients were able to communicate with the parents and research team if they experienced any pain or discomfort either due to the spinal neuromodulation or due to the locomotor procedures. None of the patients reported any pain or discomfort during the stimulation and were blinded from the stimulation parameters at any given time during the testing procedures. While the patients could feel the stimulation pulses initially, they were unable to clearly distinguish between intensities or even the presence of stimulation after a brief period of accommodation. The stimulation intensities used for each patient are listed in Table 1.

**Fig. 1:**
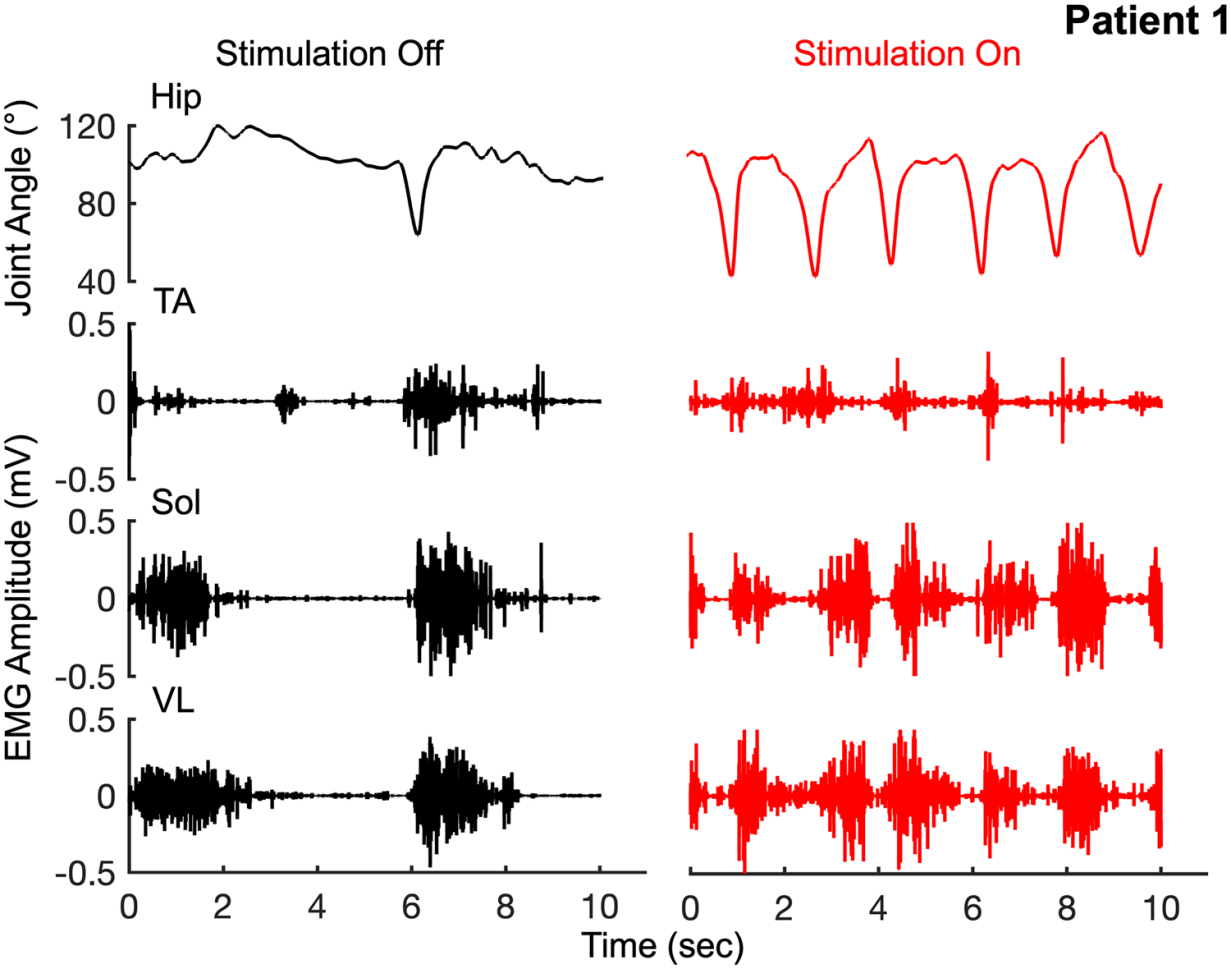
Patient no. 1 (age 2) stepping on a treadmill without and with spinal neuromodulation. Note that the patient is unable to step with the stimulation off whereas he begins to step voluntarily once the stimulation is turned on

### Data Collection

Surface electromyography (EMG) was recorded from nine out of the twelve participants from select lower extremity muscles using surface EMG electrodes (LabChart and PowerLab; ADInstruments). Electronic Goniometers were bilaterally placed on hip and/or knee joints to record joint angular displacements. Video recordings were completed in all 12 patients and video plus EMG in 5 patients. EMG recordings were not performed in 3 patients (P5, P7, and P10) due to the visible apprehension seemingly attributable to the size of the recording electrodes and wires as well as their low level of postural stability. Data were recorded and sampled at a frequency of 10 kHz and were analyzed using LabChart software. Video data were recorded on LabChart and synchronized with the EMG files.

### Locomotor Assessment

For patients who were unable to perform functional tasks such as standing or stepping, the primary outcome was the ability to voluntarily initiate these activities without any external assistance. For patients who were capable of stepping on a treadmill, the primary outcome was to assess EMG and kinematic patterns representing improved coordination and reduced co-contractions. Each patient was tested on a treadmill at the same speed and body weight support (patient 1 and 5) without and with stimulation. The speed of the treadmill was set to ensure patients were capable of stepping at a comfortable speed. Since patients 7 and 10 were unable to hold their head up or stand upright without external support, they were only tested for the ability to sit upright with the head erect and when transitioning from sit to stand.

### Data Analysis and Statistics

Joint angle excursions were calculated by identifying the stance and swing phases. Integrated EMG data are reported by calculating the area under the curve of the filtered, rectified signal for each step cycle. To calculate the level of co-contraction, we quantified the joint probability distribution (JPD) plots of all data points (resolution 0.1 ms) from two antagonistic muscles (TA and Sol) of the filtered EMG during a series of 15 consecutive step cycles. These data points were divided into 4 quadrants with the axes drawn at 20% of the max amplitude (of the linear envelope of rectified EMG trace) for each muscle. The total number of data points in quadrants 2 and 4 representing alternating activity between antagonistic muscles were summed and compared between stimulation off vs on. The coefficient of correlation was calculated by measuring the correlation between joint angles of each normalized step cycle with the normalized mean step cycle as previously published [32]. Paired t-tests were used to compare the group mean data without and with stimulation. All statistical significances are reported at *P* < 0.05.

## Results

**Fig. 2:**
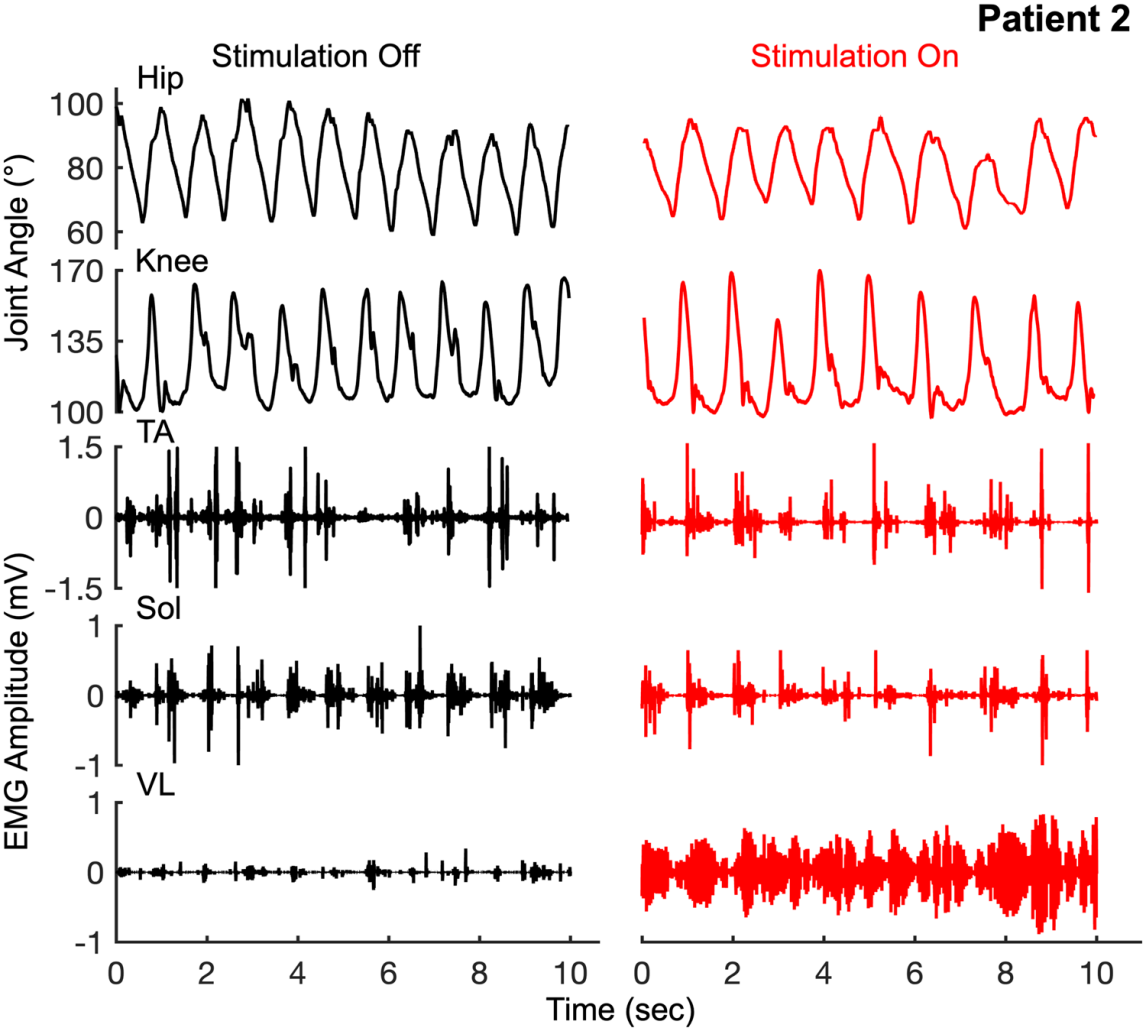
Patient no. 2 (age 7) stepping on a treadmill without and with spinal stimulation

**Fig. 3:**
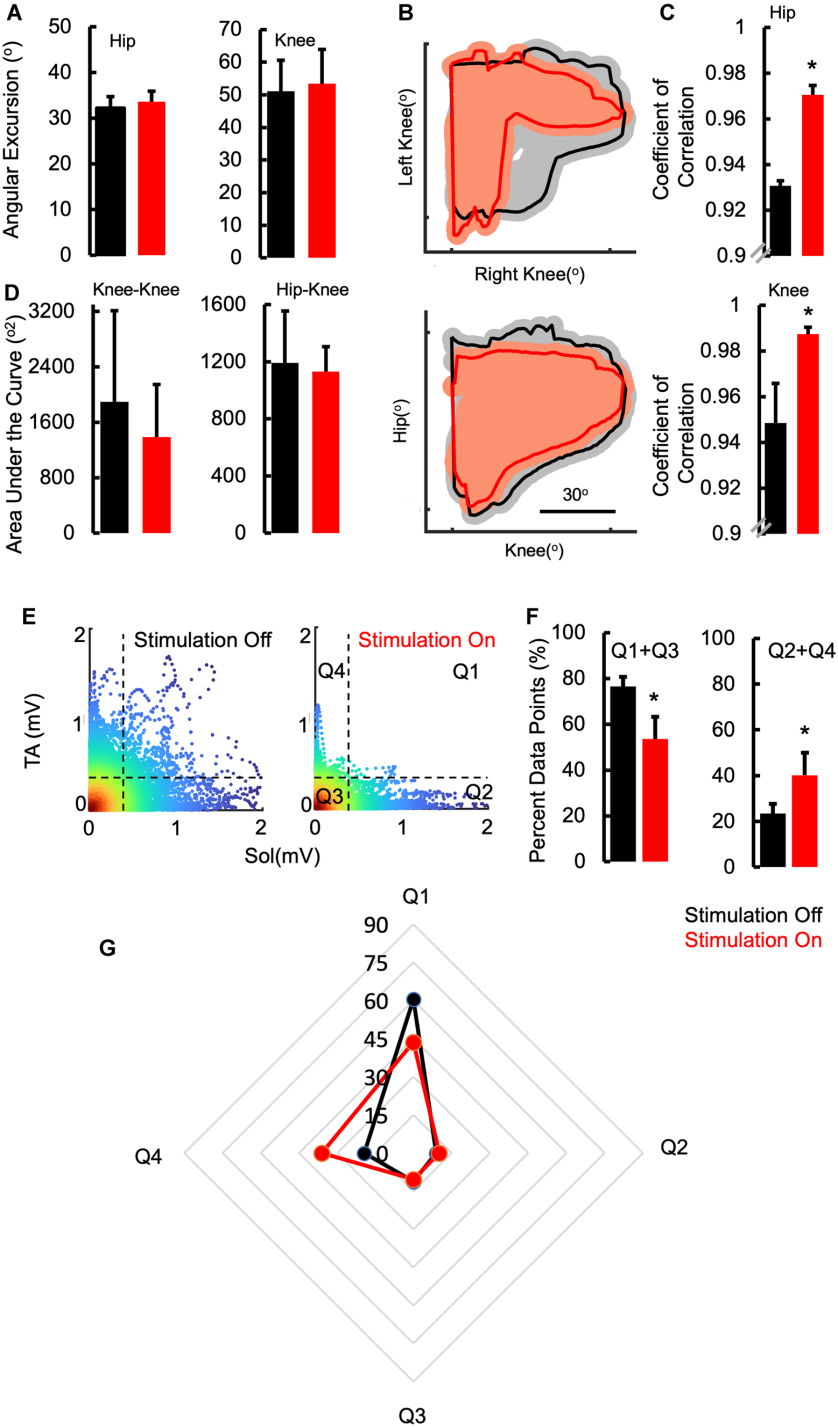
**A** Mean ± SE (n = 9 patients) angular excursion of the hip and knee joints without and with stimulation. **B** Interlimb coordination has shown from a representative patient (P2, n = 15 step cycles) by the knee-knee joint angle plots and intralimb coordination shown by the hip-knee plots without and with stimulation. Note the shaded area represents the variation over the 15-step cycle. **C** Mean ± SE (n = 9) coefficient of correlation of the trajectories shown in **B** with respect to the mean trajectory. **D** Mean ± SE (n = 9) area under the curve calculated for the plot in **B**. **E** Joint probability density (JPD) distribution plot of filtered rectified EMG amplitudes of the TA vs the Sol muscles without and with stimulation derived from an average of 15 step cycles for a representative patient (P2). **F** Mean ± SE (n = 9) percent data points in Quadrants (1 + 3) and (2 + 4) without vs with stimulation and **G** the mean percent data points for each quadrant. *Statistically significant at *P* < 0.05

The patients were first tested without stimulation for either stepping on the treadmill at their comfortable speed, sitting upright, or transitioning from sitting to standing. After a brief rest, they were then tested with stimulation at T11–12 and L1–2 for the same tasks. The intensity of the stimulation was unknown to the patient and was maintained at a sub-motor threshold intensity [30]. Patient P1 was unable to step on the treadmill without stimulation and demonstrated occasional and uncoordinated bursts in his lower extremity muscles. He could however step bilaterally with stimulation on with robust alternating EMG bursting activity in all his lower extremity muscles (Fig. 1, Supplementary video 1). Patients (P2-P6, P8, P9, P11, and P12) were capable of stepping without stimulation and demonstrated consistent bursting activity in all lower extremity muscles with significant levels of co-contraction of agonist-antagonistic muscles (Fig. 2, Supplementary Fig. 2). The overall kinetics and kinematics represented by the change in EMG activity and joint angle excursions were similar between stimulation off and on (Fig. 3A). A key difference was noted in the interlimb and intralimb coordination and the consistency of stepping as reflected in the increased coefficient of correlation with fewer variations in joint angles (hip and knee) between consecutive cycles with stimulation on vs off (*P* < 0.05) (Fig. 3B, C). While the area under the curve for joint angles (hip-knee and knee-knee) showed a trend toward a decrease with stimulation on vs off; these changes were not significant (Fig. 3D), perhaps, because the overall angular excursions remained the same (Fig. 3A). However, the patients were able to take longer steps in the presence vs absence of stimulation (0.88 ± 0.005 s vs 0.973 ± 0.036 s, *P* < 0.05). In addition, antagonistic muscles (TA vs soleus) showed significantly lower levels of co-contraction (Fig. 3E). The mean percent data points representative of alternating activity (Quadrant 2 + 4) was significantly higher with stimulation on vs off representing a decreased level of co-contraction between antagonistic muscles, a representation of decreased spasticity as well as considerably improved levels of coordination of agonist-antagonistic motor pools (Fig. 3E, F, and G). Patient 7 who was only tested for upright sitting and standing due to complete lack of head control was capable of performing the functional tasks for longer periods of time with increased levels of control (10–12 s vs 45–60 s) with stimulation on compared to off (Supplementary video 2).

## Discussion

**Fig. 4:**
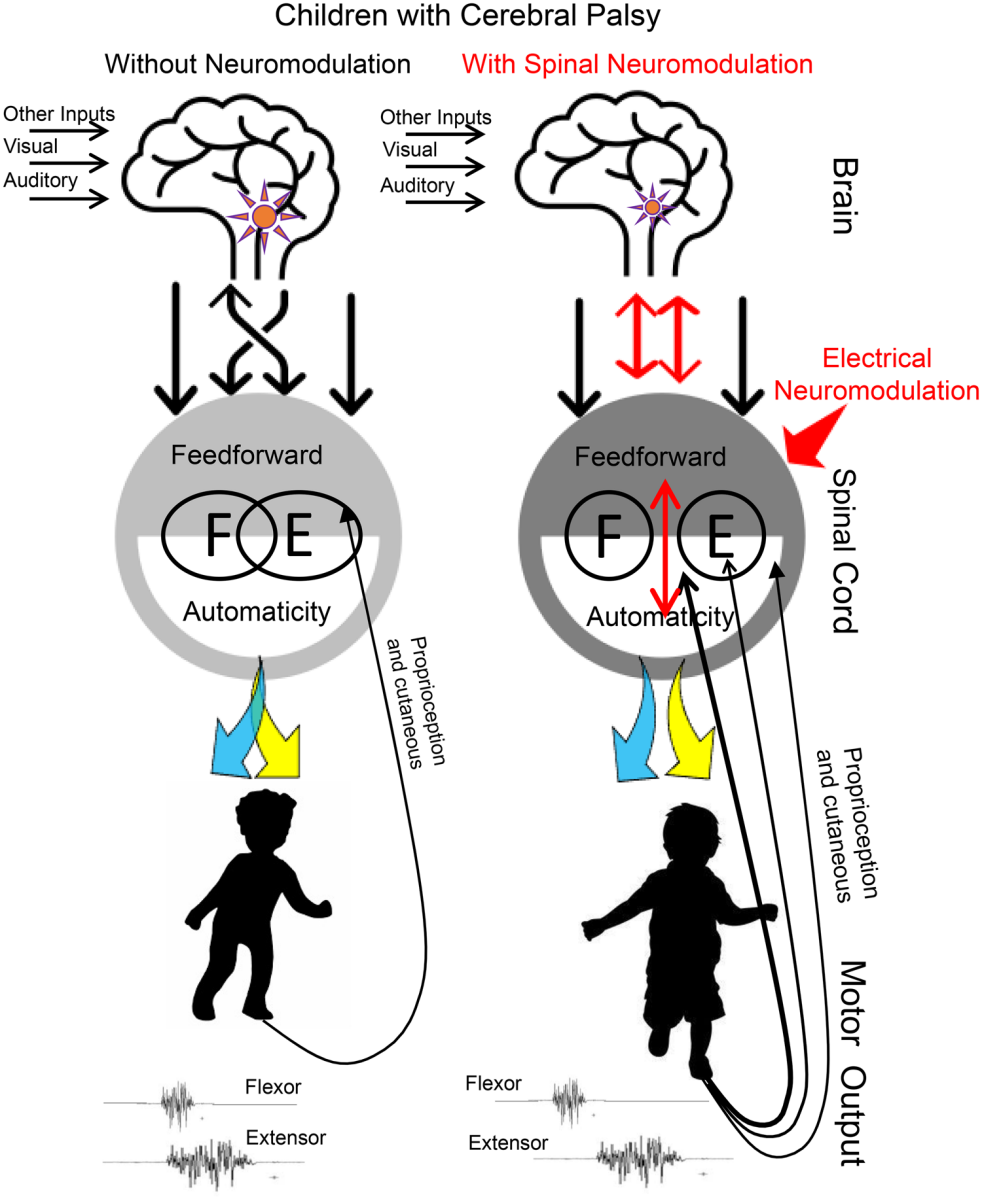
Hypothetical schematic of the brain, spinal cord, and muscles in children with cerebral palsy without and with acute spinal neuromodulation. F flexor motor pool, E extensor motor pool. Note the symbolic reduced lesion size to reflect a lesser dysfunctional supraspinal impact during spinal neuromodulation

The present data suggest that the supraspinal connectivity to the spinal neural networks in individuals with CP has developed into an incomplete and/or abnormal physiological state [33, 34]. It seems rather clear that the abnormality in the brain sends disruptive signals to the spinal networks that, in turn, generates poorly coordinated movements. We hypothesized that this abnormal supraspinal-spinal loop can be functionally minimized, largely via proprioception projecting normal signals to spinal networks enabling the automaticity and feedforwardness [35] of the spinal cord which translates the sensory input into a more normal motor action (Fig. 4) [36]. Finally, we predict that this more normal action derived from proprioception in the presence of spinal neuromodulation can be learned via activity-dependent changes (rehabilitation). One could question, or at least be surprised, as to how the outcomes of the interventional strategies used for CP are similar to those observed with spinal cord injury (SCI). Both dysfunctions, however, have evolved from abnormal spinal-supraspinal connections, but both can be acutely driven toward normality by proper posture, proprioception, and spinal neuromodulation [15–17, 19, 36–38].

CP patients demonstrate improper and perhaps incomplete functional connectivity between the brain and the spinal cord (Fig. 4). Since a relatively “normal” level and target-specific connectivity is needed to accommodate a 1-G environment, the dysfunctional brain-spinal cord connectivity results in the patient learning an abnormal gait and posture to accommodate gravity. Spinal neuromodulation changes the physiological states and the functional connections of the brain and spinal networks. The present observations of patients with CP over a wide range of age groups (2 to 50 years) and severity of dysfunction (GMFCS level I to V) are important, first, because the similarities at systems-level mechanisms of acutely reorganizing spinal-supraspinal networks generated improved coordination of motor pools in stepping are similar to that observed with SCI for multiple motor functions. Secondly, the similarities of the acute effects of both SCI and CP provide strong evidence that similar mechanisms could be relevant in achieving long-term neuroplasticity with the repetitive presentation of similar interventional parameters [36]. The acute changes in coordination of motor pools and reduced variability in stepping reported here provide strong evidence that noninvasive spinal neuromodulation can lead to positive chronic effects. It is interesting to note that the greatest improvements in functional performance during acute spinal neuromodulation were observed in either the youngest individuals or ones that were most severely affected (GMFCS IV and V) whereas the oldest reported minimal change in sensation and performance with a single exposure to neuromodulation.

The importance of these results is that it provides a lead to a novel strategy with a strong biological logic that might make it possible to realize a transformation similar to that which is occurring with spinal cord injury. Thus, we hypothesized that spinal neuromodulation in concert with proprioceptive-driven activity-dependent mechanisms of spinal networks can transform the supraspinal-spinal dysfunctional connectivity of CP into highly functional connections, characterized by a more normal agonist–antagonist coordination pattern. Unlike traditional approaches (such as functional electrical stimulation, which are designed to directly induce muscle contractions), spinal neuromodulation can be used to modulate the excitability of spinal networks below the motor threshold to a more functional physiological state when engaged primarily by more normal patterns of proprioception. We propose that these continuous ensembles of proprioception can be translated automatically with considerable precision by the spinal networks. Based on the more normal signals generated by these spinal networks. With repetitive treatments, more normal signals are projected supraspinally to movement control centers in the brain, which further restores [23] sensorimotor functions (Fig. 4). Although improvement of multiple behaviors derived from supraspinal networks following spinal injury has been clearly manifested physiologically, the spinal-supraspinal networks through which this effect is mediated have only recently become of great importance. In essence, transcutaneous spinal neuromodulation appears to facilitate neuroplasticity and can lead to bidirectional transformations of neural connections among brain-spinal cord-muscle-spinal cord-brain connectomes [39].

One of the most common spontaneous perceptions expressed by patients with CP (including the ones reported here) when initially receiving epidural or transcutaneous spinal neuromodulation, regardless of whether it was an individual with a SCI, stroke, or CP and regardless of the task being attempted, was that it was “easier” to perform and was able to perform tasks for longer periods of time with less fatigue. Another self-reported comment has been an increase in level of awareness and “sensation of stepping,” “feeling lighter” while walking on the treadmill with stimulation. Interestingly, in the first published report by Herman and colleagues in 2002 [40] and subsequently by Harkema and colleagues in 2011 [28], exploring the possibility of restoring motor function after a severe spinal cord injury, the patient’s perception was that it was easier to walk in the presence of epidural stimulation. The mechanisms by which the widely different means of neuromodulation are applied and the types of patients with different dysfunctions and the regaining of an array of sensation need to be further explored more quantitatively. There has been strikingly little effort to attempt to understand the mechanisms of recovering sensations, although there are increasingly important clues as to its fundamental importance in recovering motor function. It is also important to emphasize that while the brain-spinal cord re-connectivity can be functionally (behaviorally) substantial, the anatomical equivalent is completely unknown.

Clinically, these results suggest that the supraspinal and spinal networks are highly responsive to activity-dependent mechanisms that help drive more aligned posture, particularly when administered in concert with spinal neuromodulation [41] [42, 43]. We propose that acute spinal neuromodulation in children with CP can enable a persistent drive for functionally useful reorganization of spinal and supraspinal networks to emerge, similar to that which has been reported for stepping [31], standing [15], upper limb [16, 44], trunk stability [17], normalization of blood pressure function [45], breathing [18], bladder, bowel, and sexual function [19, 20, 46]. These observations have occurred in almost every patient who had been spinally injured for at least 1 year with complete paralysis before receiving spinal neuromodulation and some form of activity-dependent intervention. It is interesting to note the similarity in response between CP and SCI patients considering the difference in pathologies. This is supported by the facts that the electrophysiological basis of our approach is similar, i.e., (1) enabling the proprioceptive system to drive motor function and (2) re-establish appropriate connections between spinal and supraspinal centers. The results observed in CP may be further enhanced considering a stronger anatomical connection between brain and spinal cord. However, the apparent problem with the greater connectivity in CP compared to SCI is the high degree of functionally aberrant supraspinal-spinal connections, given the spastic-like behavior and the abnormal EMG patterns (Capellini et al., 2020 and Edgerton et al., 2021).

While spinal neuromodulation has been explored in children with CP, these data are unique as these is the first to demonstrate the electrophysiological changes possible within one session of stimulation to reorganize a nonfunctional or dysfunctional brain-spinal network to a new functional physiological state. In addition, it lays the foundation for when these tasks are performed repetitively, a learning phenomenon can emerge, which facilitates regaining a range of movements requiring greater skill, power, and endurance. Also, the present observations are consistent with the neuromodulation changing the physiological states of spinal and supraspinal networks in a way that enables the nervous system to choose from an infinite number of potential movements. In essence, this enables the person with CP to engage strict guidance from proprioceptive input as occurs normally, to drive the spinal networks [47].

We emphasize this latter point because the more effective spinal neuromodulation strategy after SCI has been to use below motor threshold levels of neuromodulation, as much as possible to *enable* the patient to perform a wide range of movements, by choice, not by electrically induced movements defined by the experimenter or the technical device used [43]. Supra-motor threshold levels of stimulation reduce the control by the spinal interneuronal networks, the sites at which most of the coordination of motor pools and spinal learning normally occurs. The acute reduction in co-contraction between antagonistic muscles with neuromodulation was observed in all patients. This acute effect highlights the potential importance of engaging activity-dependent mechanisms to minimize the impact of functionally aberrant connections that may be formed in the brain and spinal cord [48, 49]. In addition, it highlights the need for more long-term activity-based neurorehabilitation therapy especially for the older individuals for whom there may be a greater persistence of aberrant connections which often lead to persistent spasticity, a manifestation of poor coordination.

## Supplementary Information

Below is the link to the electronic supplementary material.

Supplementary file1 (DOCX 12 kb)

Supplementary file2 (TIFF 21471 kb)

Supplementary file3 (TIFF 28587 kb)

Supplementary file4 (MP4 25908 kb)

Supplementary file5 (MP4 64083 kb)

## Declarations

### Disclaimer

The funders were not involved in the study design, collection, analysis, interpretation of data, the writing of this article or the decision to submit it for publication.
