# Supplementary material for: Transcutaneous Spinal Neuromodulation Reorganizes Neural Networks in Patients with Cerebral Palsy
Source: Neurotherapeutics. 2021 Jul 9;18(3):1953–62. doi: 10.1007/s13311-021-01087-6 (PMC8608961; doi:10.1007/s13311-021-01087-6)
Supplement: Supplementary file 1 — Supplementary file1 (DOCX 12 kb) [file 13311_2021_1087_MOESM1_ESM.docx]

***Author Disclosure Statement:***

V.R.E, holds shareholder interest in NeuroRecovery Technologies and hold certain inventorship rights on intellectual property licensed by The Regents of the University of California to NeuroRecovery Technologies and its subsidiaries.​

V.R.E and PG holds shareholder interest in SpineX Inc. and hold certain inventorship rights on intellectual property licensed by The Regents of the University of California to SpineX Inc.
