# Supplementary figures and images for: Transcutaneous Spinal Neuromodulation Reorganizes Neural Networks in Patients with Cerebral Palsy
Source: Neurotherapeutics. 2021 Jul 9;18(3):1953–62. doi: 10.1007/s13311-021-01087-6 (PMC8608961; doi:10.1007/s13311-021-01087-6)

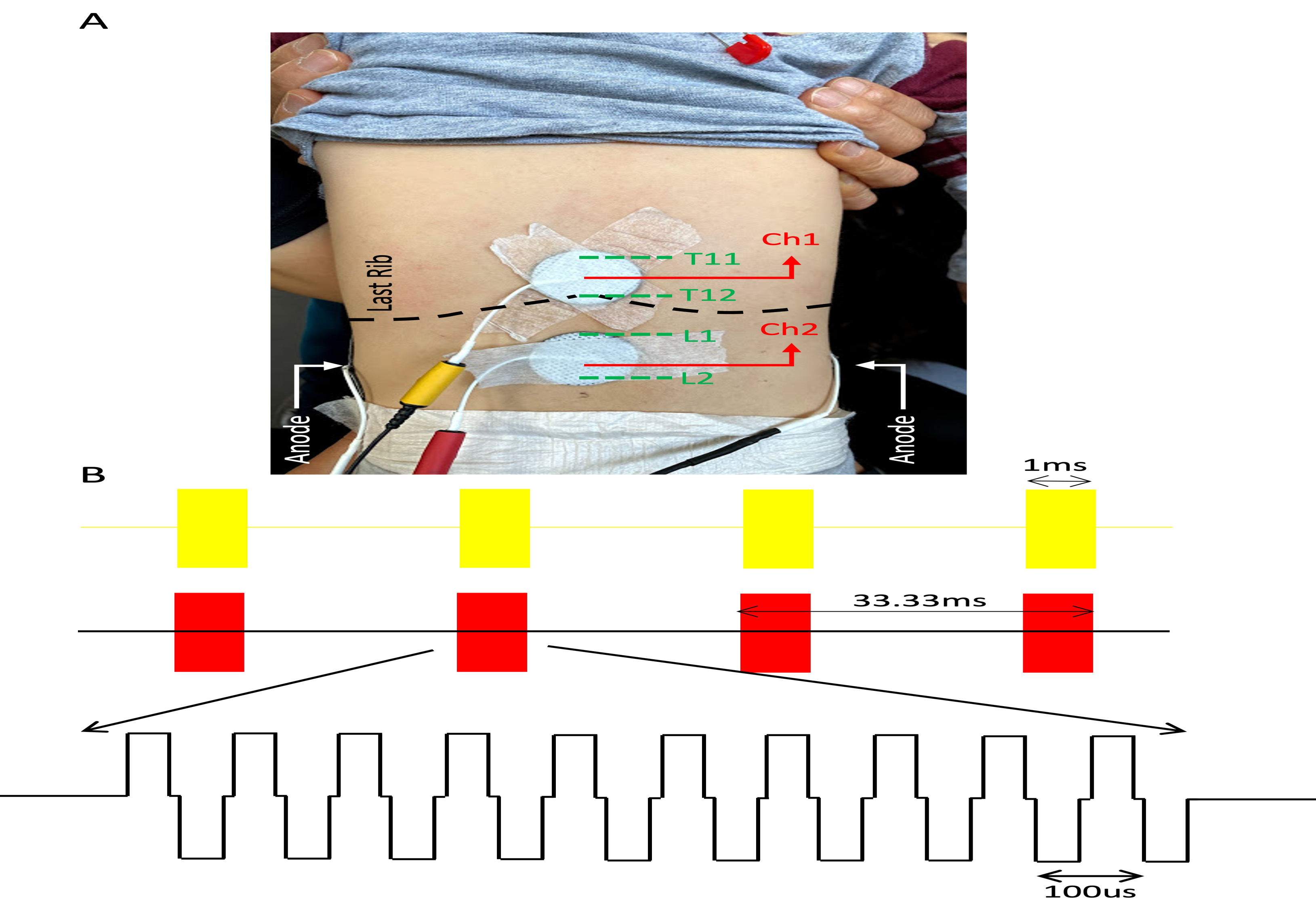

Supplement: Supplementary file 2 — Supplementary file2 (TIFF 21471 kb) [file 13311_2021_1087_MOESM2_ESM.tiff]

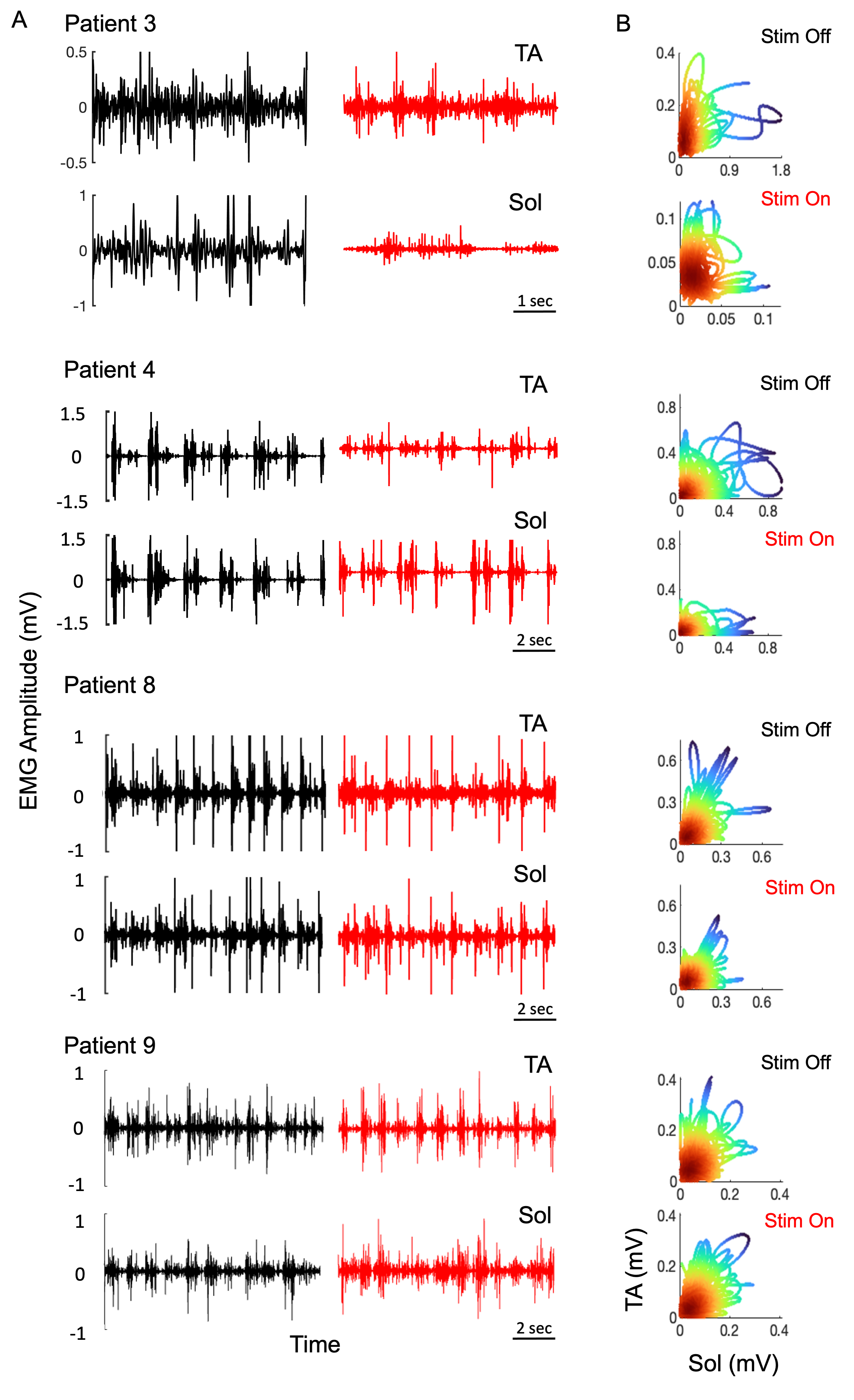

Supplement: Supplementary file 3 — Supplementary file3 (TIFF 28587 kb) [file 13311_2021_1087_MOESM3_ESM.tiff]
